# The H_2_S Donor Sodium Thiosulfate (Na_2_S_2_O_3_) Does Not Improve Inflammation and Organ Damage After Hemorrhagic Shock in Cardiovascular Healthy Swine

**DOI:** 10.3389/fimmu.2022.901005

**Published:** 2022-06-16

**Authors:** David Alexander Christian Messerer, Holger Gaessler, Andrea Hoffmann, Michael Gröger, Kathrin Benz, Aileen Huhn, Felix Hezel, Enrico Calzia, Peter Radermacher, Thomas Datzmann

**Affiliations:** ^1^ Institute for Anesthesiologic Pathophysiology and Process Engineering, Ulm University, Ulm, Germany; ^2^ Department of Transfusion Medicine and Hemostaseology, Friedrich-Alexander University Erlangen-Nuremberg, University Hospital Erlangen, Erlangen, Germany; ^3^ Department of Anesthesiology, Intensive Care Medicine, Emergency Medicine and Pain Therapy, German Armed Forces Hospital Ulm, Ulm, Germany; ^4^ Department of Anesthesiology and Intensive Care Medicine, University Hospital Ulm, Ulm, Germany

**Keywords:** physical injuries, hemorrhage, systemic inflammation, hydrogen sulfide, animal model, gaseous mediator

## Abstract

We previously demonstrated marked lung-protective properties of the H_2_S donor sodium thiosulfate (Na_2_S_2_O_3_, STS) in a blinded, randomized, controlled, long-term, resuscitated porcine model of swine with coronary artery disease, i.e., with decreased expression of the H_2_S-producing enzyme cystathionine-γ-lyase (CSE). We confirmed these beneficial effects of STS by attenuation of lung, liver and kidney injury in mice with genetic CSE deletion (CSE-ko) undergoing trauma-and-hemorrhage and subsequent intensive care-based resuscitation. However, we had previously also shown that any possible efficacy of a therapeutic intervention in shock states depends both on the severity of shock as well as on the presence or absence of chronic underlying co-morbidity. Therefore, this prospective, randomized, controlled, blinded experimental study investigated the effects of the STS in cardiovascular healthy swine. After anesthesia and surgical instrumentation, 17 adult Bretoncelles-Meishan-Willebrand pigs were subjected to 3 hours of hemorrhage by removal of 30% of the blood volume and titration of the mean arterial pressure (MAP) ≈ 40 ± 5 mmHg. Afterwards, the animals received standardized resuscitation including re-transfusion of shed blood, fluids, and, if needed, continuous i.v. noradrenaline to maintain MAP at pre-shock values. Animals were randomly allocated to either receive Na_2_S_2_O_3_ or vehicle control starting 2 hours after initiation of shock until 24 hours of resuscitation. The administration of Na_2_S_2_O_3_ did not alter survival during the observation period of 68 hours after the initiation of shock. No differences in cardio-circulatory functions were noted despite a significantly higher cardiac output, which coincided with significantly more pronounced lactic acidosis at 24 hours of resuscitation in the Na_2_S_2_O_3_ group. Parameters of liver, lung, and kidney function and injury were similar in both groups. However, urine output was significantly higher in the Na_2_S_2_O_3_ group at 24 hours of treatment. Taken together, this study reports no beneficial effect of Na_2_S_2_O_3_ in a clinically relevant model of hemorrhagic shock-and-resuscitation in animals without underlying chronic cardiovascular co-morbidity.

## Introduction

Sodium thiosulfate, Na_2_S_2_O_3_, (STS) is an H_2_S donor with minimal side effects and clinically approved for the treatment of calciphylaxis, *cis*-Pt toxicity, and cyanide poisoning (1). Along with its sulfide releasing properties it is a known antioxidant. Moreover, STS was shown to be organ-protective in rodent models of acute liver injury (2), endotoxemia (3, 4), bacterial sepsis (3, 5, 6), and, in particular, ischemia/reperfusion (I/R) injury of the brain (7), heart (8–13), and the kidney (14, 15). Organ protective properties had also been demonstrated in larger species, i.e., canine tourniquet-induced limb ischemia and myocardial infarction induced by ligation of the left anterior descending coronary (16). However, none of these models integrated standard intensive care measures into the experimental design, and, moreover, STS was mostly administered as a pre-treatment or virtually simultaneously with the initiation of ischemia.

Hemorrhagic shock and subsequent resuscitation trigger a systemic inflammatory response due to the tissue oxygen deficit and reperfusion (17–21). Therefore, we have recently tested the therapeutic potential of STS using a post-treatment approach in a blinded, randomized, controlled, long-term, resuscitated porcine model of swine with coronary artery disease (22). STS treatment showed marked lung-protective properties, whereas no effects were observed in other organs. We confirmed these beneficial effects of post-treatment STS by attenuation of lung, liver and kidney injury in mice with genetic CSE deletion (CSE-ko) undergoing trauma-and-hemorrhage and subsequent intensive care-based resuscitation (23). However, we had previously shown that any possible efficacy of a therapeutic intervention in shock states not only depends on the severity of shock *per se* (24, 25), but also on the presence or absence of chronic underlying co-morbidity, e.g., atherosclerosis (26, 27), COPD (28), or metabolic derangements (29). In fact, the above-mentioned porcine study on organ-protective effects of STS in porcine hemorrhage-and-resuscitation (22) investigated swine with coronary artery disease, and, hence, decreased expression of the H_2_S-producing enzyme cystathionine-γ-lyase (CSE) (30), i.e. an “H_2_S-poor condition” that might render exogenous, STS-derived H_2_S supplementation particularly promising (31). Based on these considerations, the aim of this study was to investigate the impact of STS on organ function in a randomized, controlled, blinded trial using a long-term, resuscitated model of hemorrhage-and-resuscitation in pigs without preexisting diseases, i.e., without underlying chronic cardiovascular co-morbidity.

## Methods

### Animals

Ethical approval was obtained by the local Animal Care Committee of Ulm University and the Federal Authorities (Tuebingen, Germany) for Animal Research (#1341). The experimental protocol was conducted in close adherence to the European Union Directive 2010/63/EU on the protection of animals used for scientific purposes. 18 adult pigs (range body weight 50-78 kg, range age 0.9-1.4 years) were purchased from the Hôpital Lariboisière, Paris, France. Pigs were of the Bretoncelles-Meishan-Willebrand strain. This strain has reduced activity of the von Willebrand Factor (vWF), thereby mimicking the human coagulation system (24, 25, 32, 33), in contrast to the hypercoagulatory state in domestic swine strains (34). Animals were sheltered at Oberberghof, Ulm, Germany, until further use with an acclimatization period of at least two weeks. Animals were kept at a cycle of 12/12 hr light/darkness and were at least monitored daily. Housing was acclimatized at 21-22°C with a humidity of 50-60% The pigs were treated with Ivermectine twice a year. The main outcome variables were 1) kidney function as assessed by creatinine clearance and 2) the noradrenaline infusion rate needed to maintain mean arterial pressure (MAP) at baseline levels. Based on our previous experiments (22, 24, 26), a case number estimation (power 0.8, α = 0.05) had yielded a group size of n = 16 (15 animals + 1 reserve animal) with a preset interim analysis after 8 animals. Because that interim analysis suggested futility, the trial was terminated prematurely in accordance with the 3R principles.

### Instrumentation and Anesthesia

Prior to instrumentation, animals were sedated by intramuscular injection of 5 mg/kg azaperone and 1-2 mg/kg midazolam followed by an insertion of an intravenous catheter into an ear vein. After pre-oxygenation, total intravenous anesthesia was induced with 1-2 mg/kg propofol and 1-2 mg/kg ketamine followed by endotracheal intubation and placement of a gastric tube for stomach decompression. Anesthesia was maintained with 6-12 mg/kg/h pentobarbitone and 30 µg/kg buprenorphine with repeated doses of 10-20 µg/kg prior to instrumentation, prior to the initiation of hemorrhagic shock as well as every 8 hours or by signs of additional demand, such as tachycardia and/or an increase in mean arterial blood pressure. Continuous i.v. pancuronium (0.15 mg/kg/h) was used for muscle relaxation. During instrumentation and hemorrhagic shock, animals were ventilated with the following parameters: fraction of inspired O_2_ (F_I_O_2_) 0.21, positive end-expiratory pressure (PEEP) 0 cmH_2_O, tidal volume 8 ml/kg, respiratory rate 10 to 12 breaths/min adjusted to maintain arterial PCO_2_ = 35 to 40 mmHg, inspiratory (I)/expiratory (E) ratio 1:2. Animals received 10 ml/kg/h Ringer’s lactate to maintain fluid balance. A central venous catheter was inserted in the jugular vein to measure central venous pressure (CVP). A thermistor-tipped pulse contour analysis catheter was placed in the femoral artery for mean arterial blood pressure (MAP) recording and trans-pulmonary single indicator thermodilution-cardiac output (CO) measurement. In the contralateral femoral artery, a 10F catheter was inserted for rapid passive blood removal to induce hemorrhagic shock. Urine was collected by a suprapubic catheter. Body temperature was assessed by a rectal probe. Animals were kept at a temperature of 37-38°C.

### Porcine Hemorrhagic Shock and Thiosulfate Regimen

**Figure 1:**
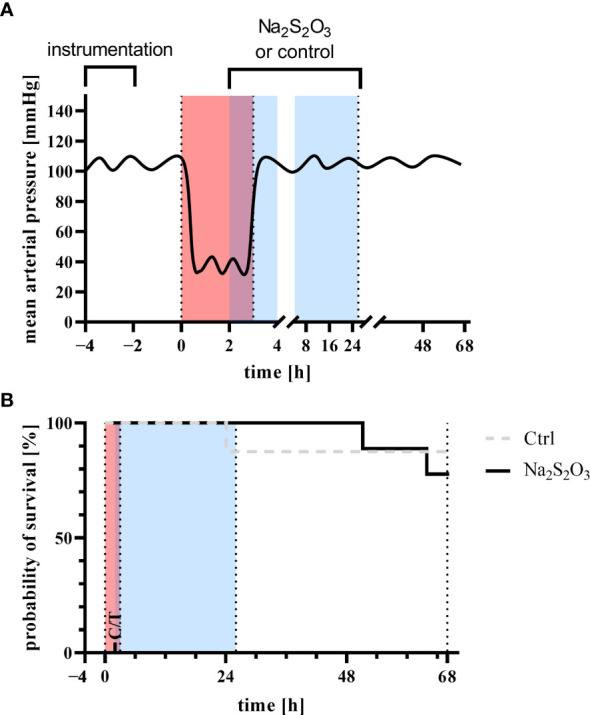
**(A)** Experimental setup and **(B)** survival analysis Log-rank Kaplan-Meyer survival analysis of animals receiving sodium thiosulfate (Na_2_S_2_O_3_, n = 9) or vehicle control (Ctrl, n = 8) for the experiment (p = 0.66, Mantel-Cox test). Red indicates the phase of hemorrhagic shock, blue indicates the phase of treatment with Na_2_S_2_O_3_ (STS, 0.025 g/kg/h for two hours followed by 0.1 g/kg/h for 23 h) or vehicle control. Instrumentation and resting period was during −4 h and 0 h, hemorrhagic shock with a target mean arterial pressure of 40 ± 5 mmHg during 0 h and 3 h (red), treatment with sodium thiosulfate or vehicle control during 2 h and 25 h (blue) with a total duration of observation and intensive care therapy of 68 h.

The experimental procedure and treatment regimen is summarized in **Figure 1A** and closely mimics previously described experiments (22, 24, 32, 35). Prior to the initiation of hemorrhagic shock after the instrumentation (1-2 h) and a resting period (total of 4 h), baseline data was collected (−0.5 h in **Figure 1**). Hemorrhagic shock was initiated by passive blood removal of 30% of the calculated total blood volume (calculated as bodyweight × 8.8%) with a target mean arterial pressure of 40 ± 5 mmHg for 3 h. Removal of the calculated blood volume was achieved within 30 min in all pigs. Every 15 min, 50 ml of blood were removed or retransfused to maintain target MAP.

Blood was stored in acid-citrate-dextrose solution until re-transfusion. 2 h after the initiation of hemorrhagic shock, animals were randomly allocated to either receive sodium STS (0.025 g/kg/h for two hours followed by 0.1 g/kg/h for 23 h) or the respective vehicle control. The lower initial infusion rate was chosen in order to take into account the reduced volume of distribution during and immediately after the hemorrhage phase. The higher infusion rate during the rest of the treatment phase was used in accordance with our previous study (22). During hemorrhagic shock, maintenance fluid was reduced to 100 ml/h. After the 3 h period, animals were treated by re-transfusion of shed blood, 10 ml/kg/h Ringer’s lactate, and continuous i.v. noradrenaline if necessary to achieve a mean arterial pressure with a target of 90-100% of the baseline. After hemorrhagic shock, ventilator settings were adjusted as follows: fraction of inspired O_2_ (F_I_O_2_) 0.35, positive end-expiratory pressure (PEEP) 10 cmH_2_O, tidal volume 8 ml/kg, respiratory rate 10 to 12 breaths/min adjusted to maintain arterial PCO_2_ = 35 to 40 mmHg, inspiratory (I)/expiratory, (E) ratio 1:1.5, peak airway pressure <40 cmH_2_O, and modified to I/E ratio 1:1 and PEEP 12 or 15 cmH_2_O, respectively, if the ratio of arterial O_2_ partial pressure (PaO_2_)/FiO_2_ was <300 or <200. The STS group consisted of 4/5 male-castrated/female pigs (62 kg; 54-71 kg), the vehicle control groups of 3/5 male-castrated/female animals (61 kg; 56-64 kg). The difference in sample size is explained by a drop out prior to the induction of hemorrhagic shock.

After the treatment and observation period (68 h after the induction of hemorrhagic shock), anesthesia was deepened and animals were sacrificed by an injection of potassium chloride. At the end of the experiment, immediate postmortem tissue sampling of lung and kidney was performed. The trial was terminated earlier if one of the following criteria were fulfilled: A) mean arterial pressure less than < 65 mmHg despite vasopressors (dosing limited to a heart rate of ≥ 160/min in order to prevent tachycardia-induced myocardial infarction); B) failure to sustain arterial PO_2_ > 60 mmHg and/or arterial Hb saturation > 90% despite maximum invasive ventilation (acute respiratory distress syndrome, ARDS); and C) acute anuric kidney failure with consecutive hyperkalemia (blood potassium > 6 mmol/L) and cardiac arrhythmia.

### Blood and Plasma Measurements

Hemodynamics, gas exchange (calorimetric O_2_ uptake and CO_2_ production), arterial blood gas tensions, acid-base status, glucose, lactate, creatinine, neutrophil gelatinase-associated lipocalin (NGAL), aspartate transaminase (AST), alanine transaminase (ALT), 8-Isoprostane, bilirubin, and troponin were determined as described previously (32, 35–37). In brief, blood gas analysis, glucose, and lactate levels were measured using a standard blood gas analyzer (ABL 800 Flex, Radiometer GmbH, Krefeld, Germany). Creatinine (#KIT044, BioPorto, Hellerup, Denmark), AST (#AS 1204, Randox, Crumlin, Northern Ireland), ALT (#AL 1205, Randox), 8-Isoprostane (#516351, Cayman Chemical, Ann Arbor, USA), bilirubin (#BR 2361, Randox), and troponin (#2010-4-HSP, Life Diagnostics, West Chester, USA), tumor necrosis factor (TNF, #PTA00, R&D Systems, Minneapolis, USA), interleukin 6 (IL6, #P6000B, R&D Systems), interleukin 10 (IL10, #P1000, R&D Systems), and super oxide dismutase (SOD, #S311, Dojindo Molecular Technologies, Rockville, USA) were determined as recommended by the manufacturer.

### Western Blot

Immediately after ending the experiment, postmortem heart, kidney, liver, and lung specimen were analyzed for Caspase-3 (#9661, Cell Signaling Technology, Danvers, USA), inducible nitric oxide synthase (iNOS, PA1-039, Thermo Fisher Scientific, Waltham, USA), heme oxygenase 1 (HO-1, #ADI-OSA-111, Enzo Life Sciences, Farmingdale, USA), nuclear factor of kappa light polypeptide gene enhancer in B-cells inhibitor, alpha (IκBα, #9242, Cell Signaling Technology), cystathionine-β-synthase (CBS, #14782, Cell Signaling Technology), Cystathionine-γ-lyase (CSE, #12217-1-AP, Rosemont), and glucocorticoid receptor (GCR, #3660, Cell Signaling Technology) as described previously (24, 26). Secondary antibodies were anti-rabbit (#7074, Cell Signaling Technology) and anti-mouse (#7076, Cell Signaling Technology) IgG, respectively. Anti-Actin (#sc-1615, Santa Cruz Biotechnology, Dallas, USA) and anti-Vinculin (#sc-73614, Santa Cruz Biotechnology) antibodies were used as loading controls. For quantitative analysis, the mean value of the individual gels from at least two gels for each animal was used. Expression of proteins was normalized to signals from two pigs (both female, 85 and 89 kg) of the same strains without further instrumentation.

### Data Analysis

Survival was analyzed using a Kaplan-Meyer-graph followed by Log-rank (Mantel-Cox) Test. Experimental data were considered to be non-parametric. The comparison of treatment and vehicle group was conducted by means of Mann-Whitney U test. Data is graphed in boxplots with median, 25^th^ and 75^th^ quantiles. Whiskers indicate upper and lower extremes, respectively. In the manuscript, data is reported as median in conjunction with 25^th^ quantile and 75^th^ quantile. Statistical analysis was conducted with GraphPad Prism9 (GraphPad Software, Inc., San Diego, California, USA). Because of non-paired testing and similar survival, missing data due to premature deaths does not largely affect the statistical analysis. We chose intentionally to not extrapolate missing data since the distance of measurement time points precluded to reliable the biological course of the variables.

## Results

### Survival

Survival did not significantly differ between the two groups (**Figure 1B**). In the STS group, two experiments had to be terminated early: one after 51 h due to refractory respiratory failure (ARDS) and another one after 64 h due to a sudden drop in MAP unresponsive to vasopressors infusion. In the vehicle control group, one animal had to be euthanized after 24 h due to ARDS.

### Parameters of Hemodynamics, Gas Exchange, Acid-Base Status, and Metabolism

**Figure 2:**
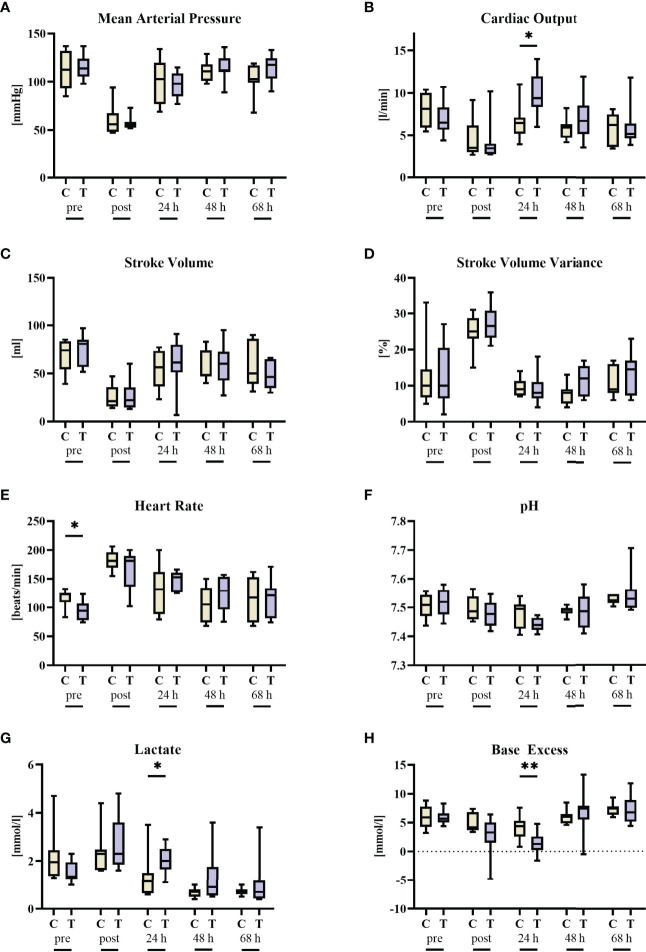
Cardiocirculatory parameters before (pre) and after 3 h of hemorrhagic shock (post) as well as 24 h, 48 h, and 68 h after resuscitation. **(A)** mean arterial pressure, **(B)** cardiac output, **(C)** stroke volume, **(D)** stroke volume variance, **(E)** heart rate, **(F)** blood pH, **(G)** blood lactate, and **(H)** blood base excess. n = 8/9 before shock, 8/9 after shock, 8/9 at 24 h, 7/9 at 48 h, and 7/8 at 68 h animals per group for vehicle control (yellow, C) and thiosulfate (purple, T), respectively. * = p < 0.05, ** = p < 0.01, Mann-Whitney U test. Box plots report median, interquartile range, minimum, and maximum.

Neither the amount of blood removed to induced hemorrhagic shock (fraction of the calculated total blood volume 39% (38; 46) vs. 32% (28; 44) in the vehicle control and STS groups, respectively, p = 0.13, Mann-Whitney U test), nor the noradrenaline infusion rates needed to achieve hemodynamic targets (0.8 µg/kg/min (0.1; 1.8) vs. 0.9 µg/kg/min (0.5; 1.1), in the vehicle control and STS groups, respectively, p = 0.81, Mann-Whitney U test) differed between the STS and vehicle group. **Tables 1**, **2** as well as **Figure 2** summarize the parameters of hemodynamics, gas exchange, acid-base status, and metabolism. There was a small but significant difference in baseline heart rate between the two groups (**Figure 2**). MAP, stroke volume, stroke volume variance, heart rate, and arterial pH did not show any significant intergroup difference, whereas cardiac output and arterial lactate levels were significantly higher and arterial base excess significantly lower in the STS group at 24 hours after shock. Of note, the peak increase of lactate within the first 24 hours after resuscitation in comparison to baseline levels measured in a 2 hour interval was 4.0 mmol/l (0.9; 4.3) in the vehicle control group and 2.8 mmol/l (1.9; 7.7) in the STS group (p > 0.99). In parallel, there was a decrease in base excess in comparison to baseline levels of −10.7 mmol/l (−16.4; 3.5) in the vehicle control group and −7.0 mmol/l (−13.0; −4.7) in the STS group (p = 0.96). Animals receiving STS had similar P_a_O_2_/F_I_O_2_ ratios and required ventilator settings to maintain target PO_2_ and PCO_2_ levels. Likewise, O_2_ uptake and CO_2_ production were similar in the two groups. Glucose levels were not affected by STS treatment either (**Table 2**).

**Table 1:** Systemic and respiratory parameters before (pre) and after 3 h of hemorrhagic shock (post) as well as 24 h, 48 h, and 68 h after resuscitation.

Parameter		pre	post	24 h	48 h	68 h
Body Temperature (°C)	C	36.0 (35.3; 36.4)	36.6 (35.7; 37.1)	37.9 (37.5; 38.2)	37.9 (37.9; 38.3)	38.1 (37.6; 38.4)
	T	35.8 (35.4; 37.0)	35.9 (35.0; 36.7)	38.2 (37.7; 38.4)	38.5 (37.8; 39.2)	38.3 (37.9; 38.6)
Central Venous Pressure (mmHg)	C	3 (1; 4)	−4 (−7; −2)	12 (9; 13)	11 (9; 17)	10 (8; 14)
	T	3 (1; 4)	−3 (−5; 1)	10 (8; 14)	11 (5; 14)	8 (4; 11)
Positive end-expiratory pressure (cmH_2_O)	C	0 (0; 0)	0 (0; 0)	10 (10; 10)	10 (10; 10)	10 (10; 12)
	T	0 (0; 0)	0 (0; 0)	10 (10; 10)	10 (10; 13)	11 (10; 13)
Respiratory Minute Volume (l × min^−1^)	C	5.0 (4.3; 5.2)	4.9 (4.3; 5.4)	6.0 (5.2; 6.3)	5.9 (4.8; 6.0)	5.8 (5.3; 7.0)
	T	4.8 (3.9; 5.4)	4.7 (3.9; 5.6)	5.7 (5.1; 6.6)	5.6 (4.7; 6.4)	6.6 (4.5; 7.3)
Arterial PO_2_ (mmHg)	C	75 (62; 88)	81 (71; 86)	127 (98; 133)	104 (89; 110)	101 (90; 119)
	T	82 (70; 89)	85 (69; 92)	124 (105; 128)	105 (75; 121)	116 (66; 125)
PaO_2_/F_I_O_2_ ratio (mmHg)	C	357 (292; 419)	387 (340; 408)	422 (326; 442)	347 (297; 367)	337 (300; 397)
	T	390 (335; 424)	404 (327; 438)	413 (340; 425)	350 (228; 402)	317 (216; 396)
Arterial PCO_2_ (mmHg)	C	37 (36; 39)	39 (34; 39)	37 (36; 40)	39 (36; 44)	37 (36; 38)
	T	35.5 (32.7; 39.9)	38.2 (31.3; 39.7)	36.4 (35.2; 40.9)	38.9 (38.1; 43.3)	37.7 (35.4; 39.2)
Oxygen Consumption (ml × min^−1^)	C	209 (197; 257)	200 (175; 236)	310 (300; 339)	308 (286; 339)	327 (284; 380)
	T	206 (188; 222)	185 (153; 227)	327 (276; 372)	361 (291; 398)	380 (301; 470)
Carbon Dioxide Elimination (ml × min^−1^)	C	159 (138; 205)	142 (139; 183)	190 (163; 221)	176 (160; 191)	184 (149; 218)
	T	145 (114; 175)	121 (108; 186)	206 (171; 225)	208 (164; 251)	202 (164; 251)

n = 8/9 before shock, 8/9 after shock, 8/9 at 24 h, 7/9 at 48 h, and 7/8 at 68 h animals per group for vehicle control (yellow, C) and thiosulfate (purple, T), respectively. Table reports median and interquartile range.

**Table 2:** Blood glucose, hemoglobin, thrombocyte and leukocyte cell count before (pre) and after 3 h of hemorrhagic shock (post) as well as 24 h, 48 h, and 68 h after resuscitation.

Parameter		pre	post	24 h	48 h	68 h
Arterial Glucose (mmol × l^−1^)	C	98 (91; 108)	109 (98; 118)	94 (72; 135)	67 (60; 83)	69 (58; 64)
	T	109 (89; 115)	108 (99; 132)	98 (83; 125)	71 (67; 84)	70 (56; 75)
Hemoglobin (g × l^−1^)	C	79 (69; 81)	93 (83; 100)	95 (84; 99)	73 (69; 92)	84 (71; 91)
	T	83 (78; 88)	93 (82; 102)	107 * (101; 116)	97 * (92; 109)	90 (82; 95)
Thrombocytes (×10^9^ × l^−1^)	C	235 (193; 271)	226 (211; 269)	221 (170; 268)	268 (129; 315)	250 (83; 330)
	T	238 (169; 340)	251 (215; 316)	248 (181; 311)	169 (112; 267)	157 (76; 222)
Leukocytes (×10^12^ × l^−1^)	C	15.8 (8.3; 18.6)	14.4 (12.4; 18.5)	14.9 (9.0; 16.0)	11.1 (7.6; 14.4)	11.6 (4.8; 18.2)
	T	13.7 (11.8; 17.2)	18.7 (14.4; 19.7)	20.5 ** (16.2; 28.4)	12.7 (11.1; 13.3)	10.2 (9.1; 16.3)

n = 8/9 before shock, 8/9 after shock, 8/9 at 24 h, 7/9 at 48 h, and 7/8 at 68 h animals per group for vehicle control (yellow, C) and thiosulfate (purple, T), respectively. * = p < 0.05, ** = p < 0.01, Mann-Whitney U test. Table reports median and interquartile range.

### Parameters of Heart, Kidney, and Liver Function and Organ Injury

**Figure 3:**
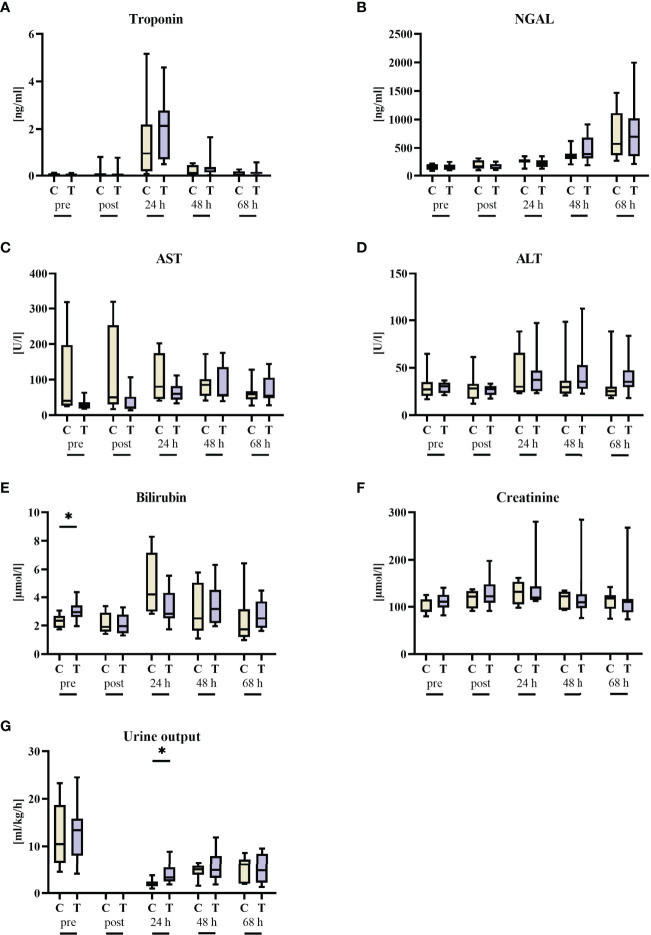
Organ function parameters before (pre) and after 3 h of hemorrhagic shock (post) as well as 24 h, 48 h, and 68 h after resuscitation. **(A)** Troponin, **(B)** neutrophil gelatinase-associated lipocalin (NGAL), **(C)** aspartate transaminase (AST), **(D)** alanine transaminase (ALT), **(E)** bilirubin, **(F)** creatinine, and **(G)** urine output. n = 8/9 before shock, 8/9 after shock, 8/9 at 24 h, 7/9 at 48 h, and 7/8 at 68 h animals per group for vehicle control (yellow, C) and thiosulfate (purple, T), respectively. * = p < 0.05, Mann-Whitney U test. Box plots report median, interquartile range, minimum, and maximum.

There were no significant intergroup differences in troponin, AST, ALT, and bilirubin (**Figure 3**). Of note, baseline bilirubin concentrations were slightly, but significantly higher in the vehicle group, which coincided with a non-significant trend towards higher AST levels in these animals. While urine output was significantly higher in the STS group at 24 h of resuscitation, parameters of kidney (dys)function (NGAL, creatinine) did not differ between the two groups.

### Inflammation

**Figure 4:**
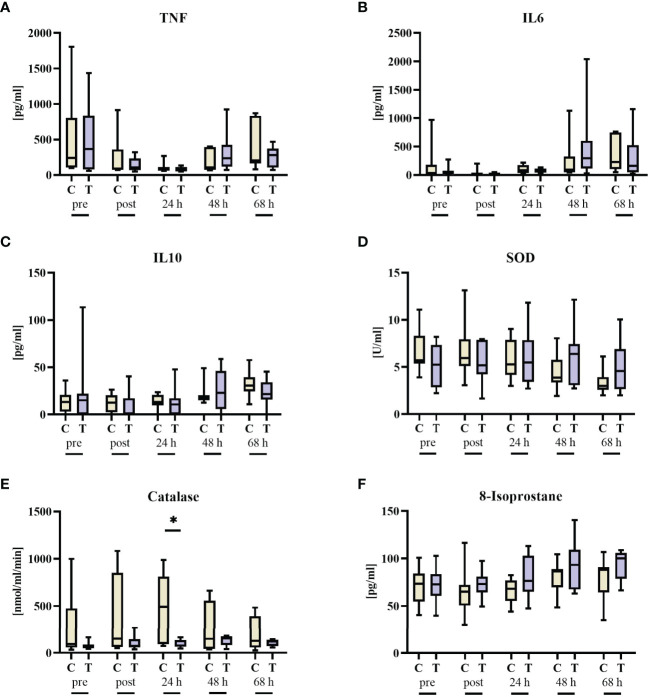
Inflammation parameters before (pre) and after 3 h of hemorrhagic shock (post) as well as 24 h, 48 h, and 68 h after resuscitation. **(A)** TNF, **(B)** IL6, **(C)** IL10, **(D)** superoxide dismutase (SOD), **(E)** catalase, and **(F)** 8-isoprostane. n = 8/9 before shock, 8/9 after shock, 8/9 at 24 h, 7/9 at 48 h, and 7/8 at 68 h animals per group for vehicle control (yellow, C) and thiosulfate (purple, T), respectively. * = p < 0.05, Mann-Whitney U test. Box plots report median, interquartile range, minimum, and maximum.

Last, the impact of STS treatment on inflammation was assessed during and after hemorrhagic shock. Animals receiving STS had significant higher leukocyte counts 24 h after hemorrhagic shock (**Table 2**). In contrast, systemic levels of TNF, IL6, IL10, 8-isoprostanes, and SOD activity were comparable in the two groups (**Figure 4**). Of note, there was a significant increase in catalase activity 24 h after shock, likely due to the pre-existing, non-significant difference already at baseline.

**Figure 5:**
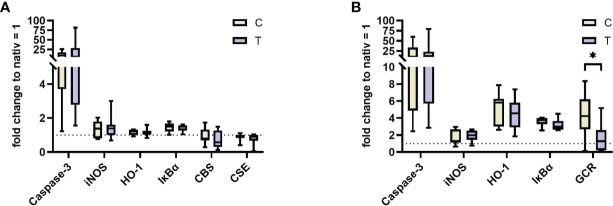
Western Blot analysis of kidney **(A)** and lung **(B)** at the end of the trial for n = 7/8 animals per group for vehicle control (yellow, C) and thiosulfate (purple, T), respectively. Results are normalized to protein levels from two untreated animals. iNOS, inducible nitric oxide synthase; HO-1, heme oxygenase 1; IκBα, nuclear factor of kappa light polypeptide gene enhancer in B-cells inhibitor, alpha; CBS, cystathionine-β-synthase; CSE, Cystathionine-γ-lyase; GCR, glucocorticoid receptor. * = p < 0.05, Mann-Whitney U test. Box plots report median, interquartile range, minimum, and maximum.

To further characterize the impact of STS on protein levels in kidney and lung, western blot analysis was conducted (**Figure 5**). No significant alterations for caspase 3, iNOS, HO-1, IκBα, CBS and CSE (the last two only analyzed in the kidney due to technical reasons) were detected. However, there was a significant decrease in GCR protein levels in the STS group. The original western blot captures are presented in **Supplementary Figures 1**, **2**.

## Discussion

Using a long-term, resuscitated, porcine model of hemorrhage-and-resuscitation, the present randomized, controlled, blinded trial was to test the hypothesis whether STS would attenuate organ dysfunction in adult animals with normal CSE expression, and, consequently, well-maintained endogenous H_2_S availability. This study therefore expands the knowledge on STS as therapeutic option in systemic inflammation in addition to “H_2_S-poor conditions”, e.g. as a result of underlying coronary artery disease (22, 30) or even genetic CSE deletion (CSE-ko) (23) that might enhance STS efficacy (31). The main findings were that *i)* STS did not beneficially affect any variable measured of hemodynamics, lung mechanics and gas exchange, or organ (dys)function and injury, nor *ii)* any of the parameters of systemic and organ inflammation or oxidative and nitrosative stress.

Possible organ-protective effects of STS have been referred to attenuated activation of nuclear transcription factor-κB, hyper-inflammation and oxidative stress (2, 3) and reduced apoptosis (9–11, 13). In addition, we had demonstrated that the marked lung-protective effect of STS in swine with coronary artery disease and, hence, reduced CSE expression coincided with significantly higher tissue glucocorticoid receptor (GR) expression. We had confirmed this finding otherwise healthy, CSE-ko mice receiving STS in addition to standard ICU care during resuscitation from trauma-and-hemorrhage (23). In sharp contrast to these previous results, lung tissue GR expression was even significantly lower in the STS-treated swine than in the vehicle group in the present study. We can only speculate regarding this different result, but the reduced activity of the von Willebrand Factor (vWF) in our Bretoncelles-Meishan-Willebrand pigs may assume importance in this context. We studied heterozygous individuals of this swine strain, because they closely mimic the human coagulation system (24, 25, 32, 33), in contrast to the hypercoagulatory state in other domestic swine strains (34). We cannot exclude, however, that this “vWF-disease”, albeit not presenting with any clinically relevant bleeding disorder, may have altered the STS effect on the GR expression: ACTH secreting bronchial carcinoid cells were shown to present with significant glucocorticoid receptor expression (38), and high glucocorticoid levels due to Cushing’s disease are associated with increased vWF-activity (39). Exogenous glucocorticoid administration produced less consistent results: in healthy subjects, oral steroids also *increased* vWF activity (40), while under hyper-inflammatory conditions the opposite effect was reported (41). To the best of our knowledge, however, no data are available on the reverse relation, i.e. on the effect of reduced vWF activity *per se* on cortisol concentrations and/or GR expression.

At the end of the treatment phase, i.e., at 24 h of resuscitation, STS was associated with statistically significantly lower arterial base excess levels (STS 1.3 mmol/l (0.3; 2.6) vs. vehicle control 4.4 mmol/l (2.6; 5.3), p < 0.01, Mann-Whitney U test). This difference in the acid-base status disappeared until the end of the experiment. The lower base excess is well in line with case reports on i.v. STS (42, 43) as well as in our previous study in swine with coronary artery disease (22). Of note, the arterial base excess tended to be lower in the STS-treated animals at the end of the shock-phase (STS 3.3 mmol/l (1.5; 5.1) vs. vehicle control 4.2 mmol/l (3.7; 6.8), p = 0.12, Mann-Whitney U test), most likely as a result of the fact that the STS-infusion was already started at 2 h of hemorrhage, i.e. 1 h prior to the initiation of re-transfusion of shed blood, fluid resuscitation, and continuous i.v. noradrenaline. Nevertheless, the fact that this fall in base excess was not associated with a pH < 7.4, any acidosis-related lung protection may have been prevented: acidosis-related lung-protection *in vivo* was reported at pH ≈ 7.00-7.25 (44).

### Limitations

We might have missed a putative therapeutic benefit due to an unbalanced shock severity: Albeit not statistically significant, the amount of blood removed to induced hemorrhagic shock, tended to be higher in the vehicle group (p = 0.13, see above, “*Results*” section). However, not only tended arterial base excess to be lower (see above), but also arterial lactatemia to be slightly higher (STS 2.3 mmol/l (1.9; 3.6) vs. vehicle control 2.3 mmol/l (1.6; 2.5), p = 0.65, Mann-Whitney U test) in the STS-treated animals already at the end of the shock phase. In addition, heart rate in the STS-group had shown a significantly higher baseline value, i.e., the relative heart increase was more severe, possibly suggesting a more pronounced activation of the sympathetic system. Clearly, any difference in shock severity was due to chance, because we used a blinded, random and controlled experimental design. Another limitation is that we might have missed a temporary effect of STS on organ function due to the distance between measurement time points, e.g., a change in organ function after 6 h of treatment. However, based on the similar survival between the groups, there is no hint for such an issue.

## Conclusion

Altogether, in contrast to our previous study in swine with coronary artery disease, this study reports no beneficial effect of STS using a blinded, randomized controlled trial design in a clinically relevant, long-term porcine model of hemorrhagic shock-and-resuscitation in animals devoid of underlying chronic cardiovascular co-morbidity. We cannot exclude that studying adult animals with heterozygous “vWF” disease may have influenced this result. Nevertheless, the current study highlights the impact of the severity of shock *per se* as well as the investigation of chronic underlying co-morbidities on the possible efficacy of therapeutic interventions in pre-clinical shock research.

## Data Availability Statement

The original contributions presented in the study are included in the article/**Supplementary Material**. Further inquiries can be directed to the corresponding author.

## Ethics Statement

The animal study was reviewed and approved by local Animal Care Committee of Ulm University and the Federal Authorities (Tuebingen, Germany) for Animal Research (#1341).

## Author Contributions

Conceptualization: PR; methodology: DM, AnH, and PR; formal analysis: DM and PR; investigation: DM, HG, AnH, MG, KB, AiH, MG, KB, FH, EC, PR, and TD, resources: PR; writing – original draft: DM and PR; writing – review and editing: DM, HG, AnH, MG, KB, AiH, FH, EC, PR, and TD; visualization: DM; supervision: PR. All authors have read and approved the final version of the article.

## Funding

DM received funding by means of a “Gerok Rotation” (rotation as clinician scientist) by the Collaborative Research Center 1149 (project number 251293561), German Research Foundation. PR received funding from the Collaborative Research Center 1149 (project number 251293561), German Research Foundation, and the German Ministry of Defense (project E/U2AD/ID013/IF564). The funders had no role in the design of this study, data collection, or interpretation, or the decision to submit the results.

## Conflict of Interest

The authors declare that the research was conducted in the absence of any commercial or financial relationships that could be construed as a potential conflict of interest.

## Publisher’s Note

All claims expressed in this article are solely those of the authors and do not necessarily represent those of their affiliated organizations, or those of the publisher, the editors and the reviewers. Any product that may be evaluated in this article, or claim that may be made by its manufacturer, is not guaranteed or endorsed by the publisher.
