# Supplementary material for: The H2S Donor Sodium Thiosulfate (Na2S2O3) Does Not Improve Inflammation and Organ Damage After Hemorrhagic Shock in Cardiovascular Healthy Swine
Source: Front Immunol. 2022 Jun 16;13:901005. doi: 10.3389/fimmu.2022.901005 (PMC9243230; doi:10.3389/fimmu.2022.901005)
Supplement: Supplementary file 2 [file DataSheet_2.docx]

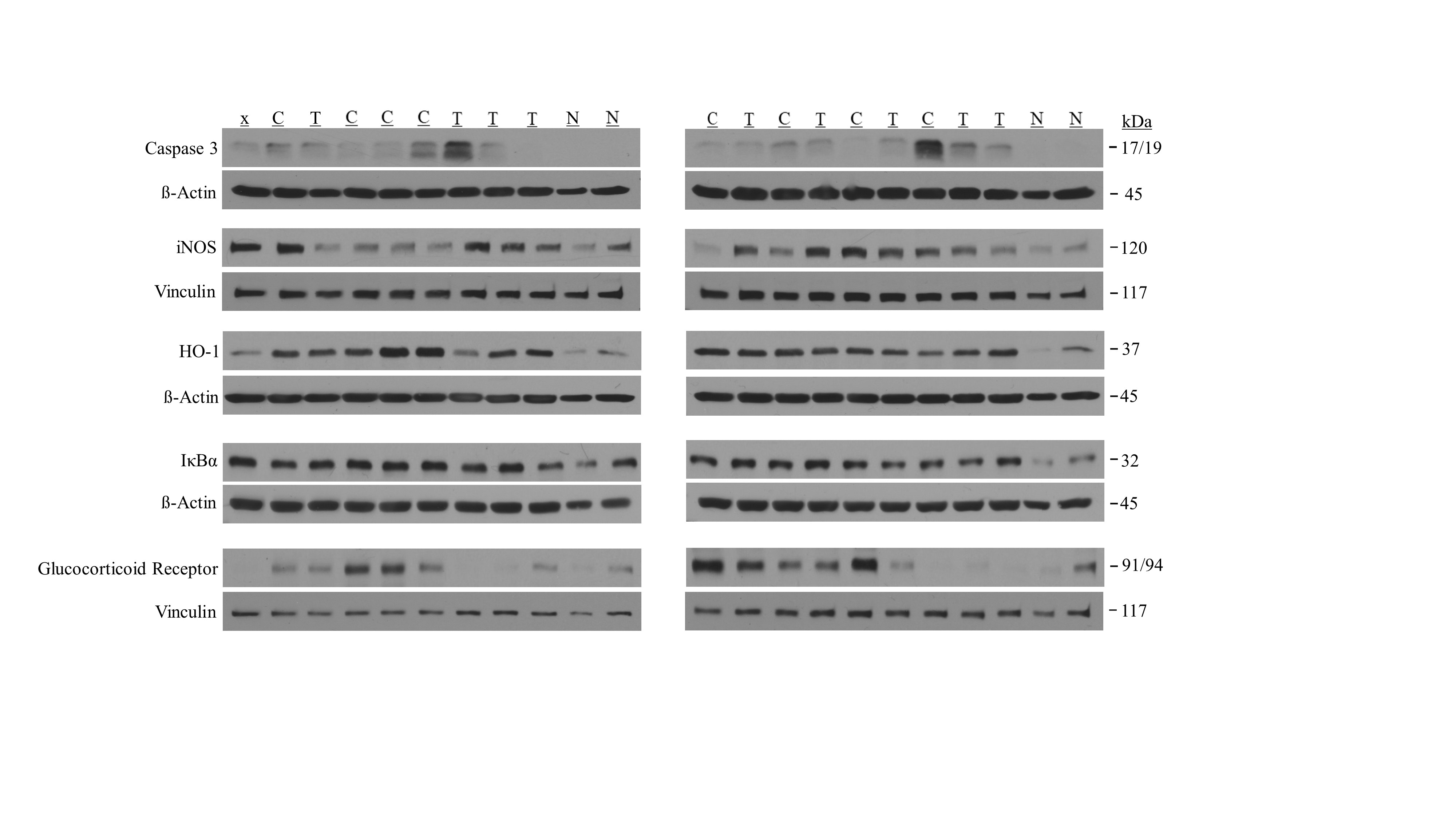


**Supplementary Figure 2.** Western Blot analysis of the lung at the end of the trial for n = 7/8 animals per group for vehicle control (C), thiosulfate (T), native animals (N), and one drop-out (x), respectively. iNOS = inducible nitric oxide synthase, HO-1 = heme oxygenase 1, IκBα = nuclear factor of kappa light polypeptide gene enhancer in B-cells inhibitor, alpha.
